# Breast-Volume Displacement Using an Extended Glandular Flap for Small Dense Breasts

**DOI:** 10.1155/2011/359842

**Published:** 2011-09-20

**Authors:** Tomoko Ogawa, Noriko Hanamura, Masako Yamashita, Hiroko Kimura, Yumi Kashikura

**Affiliations:** Department of Breast Surgery, Mie University Graduate School of Medicine, Mie 514-8507, Japan

## Abstract

We defined the glandular flap including fat in the subclavicular area as an extended glandular flap, which has been used for breast-conserving reconstruction in the upper portion of the breast. *Indication*. The excision volume was 20% to 40% of the breast volume, and the breast density was dense. *Surgical Technique.* The upper edge of the breast at the subclavicular area was drawn in the standing position before surgery. After partial mastectomy, an extended glandular flap was made by freeing the breast from both the skin and the pectoralis fascia up to the preoperative marking in the subclavicular area. It is important to keep the perforators of the internal mammary artery and/or the branches of the lateral thoracic artery intact while making the flap. *Results*. Seventeen patients underwent remodeling using an extended glandular flap. The cosmetic results at 1 year after the operation: excellent in 11, good in 1, fair in 3, and poor in 2. All cases of unacceptable outcome except one were cases with complications, and more than 30% resection of moderate or large size breasts did not obtain an excellent result for long-term followup. *Conclusion*. This technique is useful for performing the breast-conserving reconstruction of small dense breasts.

## 1. Introduction

Conservative breast surgery has rapidly become the standard procedure for breast cancer. Cancer control is the primary goal of all breast-cancer treatments, but conservative breast surgery has the additional goal of achieving cosmetic results that are acceptable to the patients. However, conservative breast surgery has not always produced good cosmetic results in all patients. The important factors considered to influence the cosmetic results are the excision volume, tumor location, and glandular density [1]. The excision volume is the most predictive factor of the cosmetic outcome, and it should be estimated in comparison to the total volume. Previous studies have suggested that excision of 20% of the breast volume has a clear risk of deformity [2]. The upper outer quadrant of the breast is a favorable location for large-volume excisions. In this location, defects can be corrected by the mobilization of adjacent tissue. In contrast, the lower pole of the breast is a less favorable tumor location [1, 3]. Glandular density can be evaluated using mammography. A dense glandular breast can be mobilized with undermining and advancement of breast tissue without complications. However, low-density breast tissue with major fatty components has a much high risk of fat necrosis if extensive undermining is performed [1].

Many Japanese females have small- to moderate-sized and dense glandular breasts. For patients with small-sized breasts, the percentage of the breast defect compared with the remainder of the breast is often large, even if the tumor size is relatively small. However, a dense glandular breast can be easily mobilized by advancing the breast tissue into the excision cavity without a risk of fat necrosis. In the upper portion of the breast, defects can be corrected by the mobilization of adjacent tissue, and furthermore, clothing that shows the décolletage is not popular in Japan, so it is possible to use adipose tissue from the subclavicular area for breast remodeling. 

 We named the mammary gland including fat in this subclavicular area as an extended glandular flap, which has been used for volume displacement for breast-conserving reconstruction in the upper portion of the dense breast. The aim of this report is to describe the efficacy of breast-volume displacement using an extended glandular flap. 

## 2. Methods

### 2.1. Subjects (Table 1)

Seventeen females underwent remodeling using an extended glandular flap after an excision of more than 20% of their breast volume (age range 30–65 years, average 45.9 years) between July in 2008 and November in 2009. All patients had a tumor in the upper portion of the breast. Fifteen patients underwent a sentinel lymph nodes biopsy. Twelve patients had no metastasis in the sentinel lymph nodes, allowing them to avoid an axillary lymph node dissection. Three patients with metastasis in the sentinel lymph nodes and 2 patients with lymph node metastasis diagnosed before the operation underwent an axillary lymph node dissection. The operation was performed by breast surgeons without the help of plastic surgeons.

The preoperative evaluation were performed by the breast surgery group. This procedure for filling of the defect was indicated for the cases whose excision volumes were 20% to 40% of the breast volume, and those breast densities were classified as heterogeneously dense or extremely dense breast tissue based on the Breast Imaging Reporting and Data System (BI-RADS) [4]. However, there were several cases that were considered to have a defect that could be compensated by the mobilization of the surrounding breast tissue before the operation, but where the operation was changed to this procedure during surgery.

The breast density of all 17 cases in this report was heterogeneously dense, and the excision volume percentage of the breast volume was 20% in 2 case, 30% in 9 cases, and 40% in 6 cases. The size of the breast was small in 11 cases, moderate in 5 cases, and large in one case.

### 2.2. Surgical Techniques

**Figure 1:**
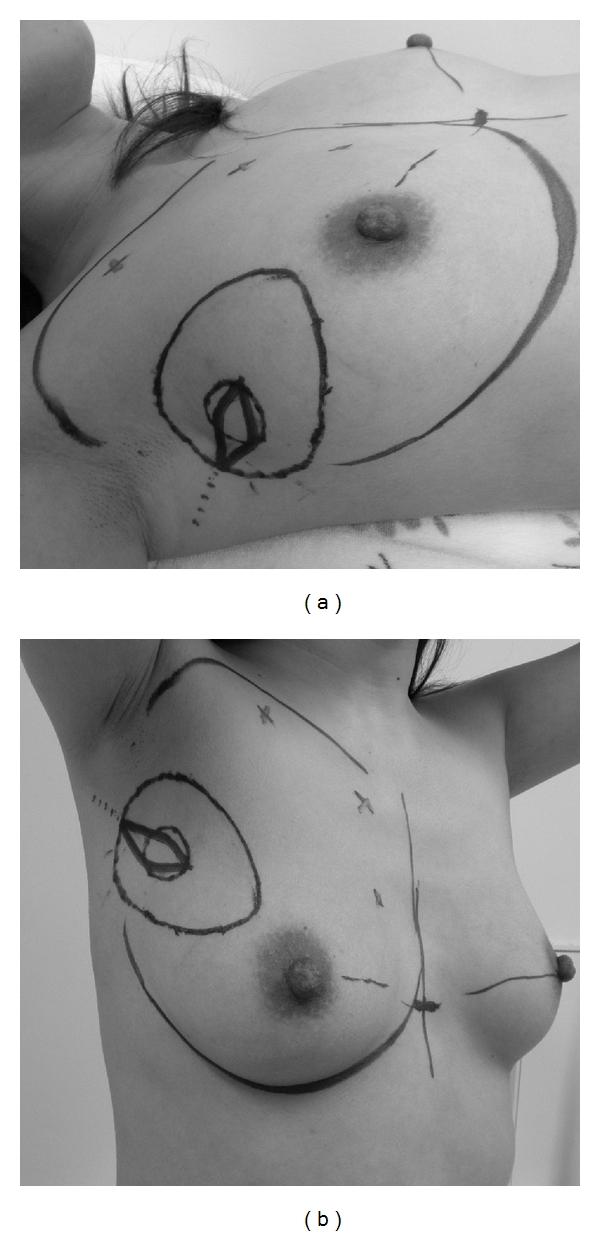
The design before the operation. Marking the partial resection area, the upper edge of the breast at the subclavicular area and the position of the nipple in the standing position, and the location of perforators from the internal mammary artery. (a) Supine position. (b) Oblique view in the standing position.

The upper edge of the breast at the subclavicular area was drawn on the skin in the standing position prior to surgery, after marking the partial resection area in the supine position. The position of the nipple in the standing position was also drawn. A doppler examination was performed to confirm the location of perforators from the internal mammary artery (Figure 1). We perform the same preoperative markings for all cases who undergo breast conserving surgery in the upper portion of the breast, because there is a possibility that a case initially thought to be compensated using only for the mobilization of the surrounding breast tissue before operation may have to be converted to this procedure during surgery. 

**Figure 2:**
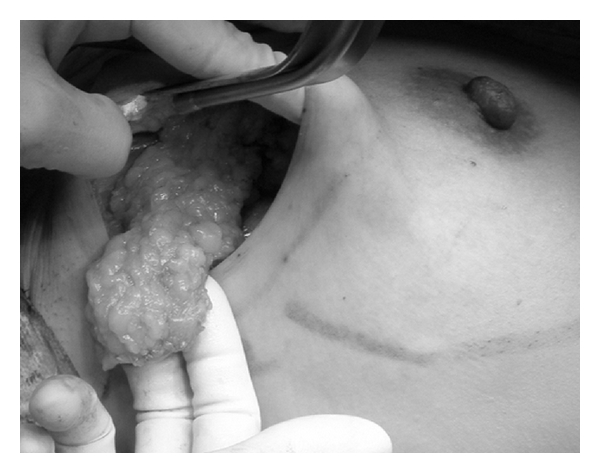
The extended glandular flap.

The breast was partially resected, and the extended glandular flap was made by freeing the breast from both the skin and the pectoralis fascia up to the subclavicular area that was marked before surgery (Figure 2). It is important to keep the perforators of the internal mammary artery intact if it is an outside tumor, and the branches of the lateral thoracic artery intact if it is an inside tumor, while making the flap.

**Figure 3:**
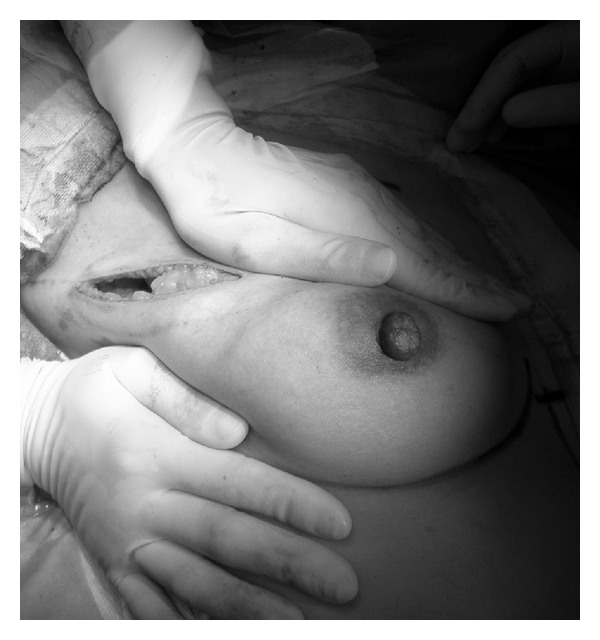
Remodeling the breast using the extended glandular flap. The nipple position is set at the position that had been marked before surgery in the standing position while applying pressure from the upper side.

**Figure 4:**
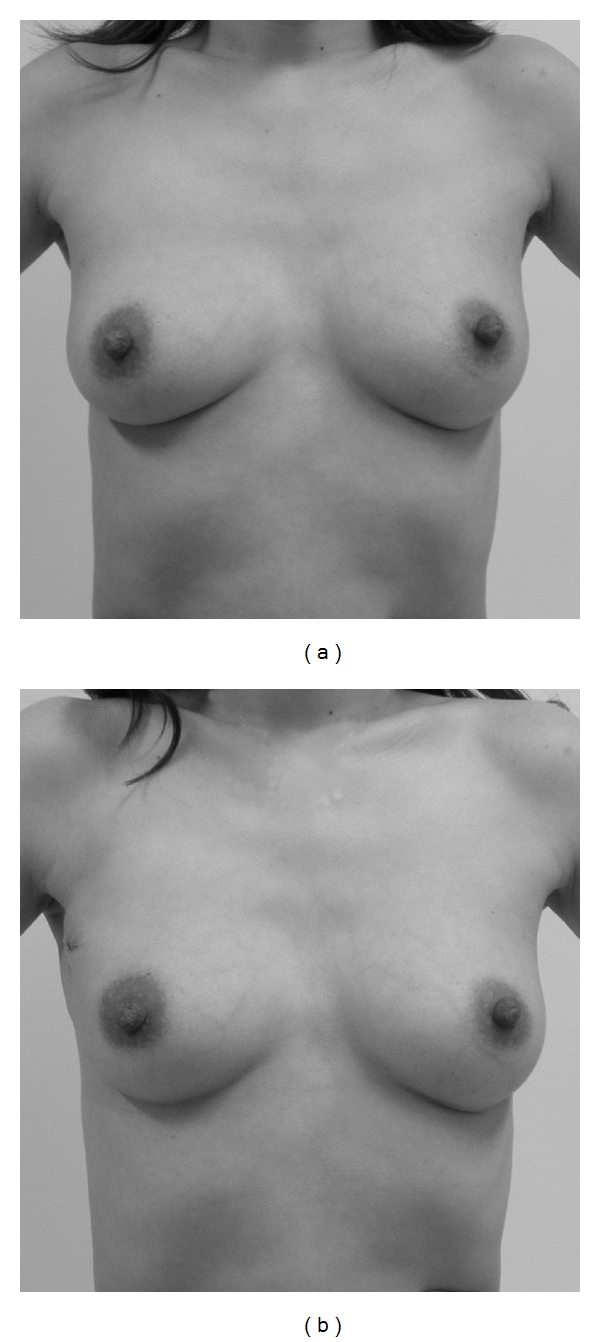
The patient of extended glandular flap. (a) Preoperative photograph. (b) Postoperative photograph at 1month after the operation.

The flap was moved to the breast area where the tumor had been removed. The flap was inserted, and the shape of the breast was checked while applying pressure from the upper side until the nipple position was set at the position that had been marked in the standing before surgery position (Figure 3), and then the flap is secured with absorbable sutures to the surrounding breast tissue. The breast was reshaped with this extended glandular flap, and skin was then sutured and a suction drain was placed in area where the skin had been freed from the underlying tissue (Figure 4). Radiation therapy was administered to the breast after wound healing, as usual.

### 2.3. Assessment of Reconstruction

The cosmetic assessment was evaluated based on photographs taken one year after the operation. Photographs of the patient's breasts were then taken using a frontal view without any identifying features. The cosmetic results were evaluated by five independent observers (breast surgeons) as “excellent”, “good”, “fair”, or “poor” using the Harvard Scale established by Harris et al. [5]. An excellent result showed the treated breast was almost identical to the untreated one, a good result: the treated breast was slightly different from the untreated breast, a fair result: there was an obvious difference between the two sides without major distortion, and a poor result: the treated breast was seriously distorted. The observers were 'blinded' to the identity of the patients. 

## 3. Results

Operative time and the blood loss of the patients without axillary dissection ranged from 95 to 134 minutes (average 111.0 minutes) and from 13 to 67 g (average 38.0 g), respectively. Operative time and the blood loss of the patients with axillary dissection ranged from 106 to 175 minutes (average 149.0 minutes) and from 24 to 125 g (average 83.8 g), respectively. Three patients had postoperative bleeding, and one of them had surgery again for bleeding and hematoma evacuation. Suction drains were placed in all cases, and the drains were removed on average 3.8 days and 8.8 days after surgery in the cases without axillary dissection and with axillary dissection, respectively. The hospital stay of the patients without and with axillary dissection ranged from 6 to 13 days (average 7.8 days) and from 10 to 14 days (average 12.6 days), respectively. These values were not statistically different from those obtained from patients who had undergone normal breast conservation therapy without extended glandular flap reconstruction. Two patients had delayed wound healing but went on to heal within 2 months. Partial fat necrosis of the glandular flap was noted in 2 patients (13.3%) (Table 1). 

### 3.1. Cosmetic Results (Table 1)

**Figure 5:**
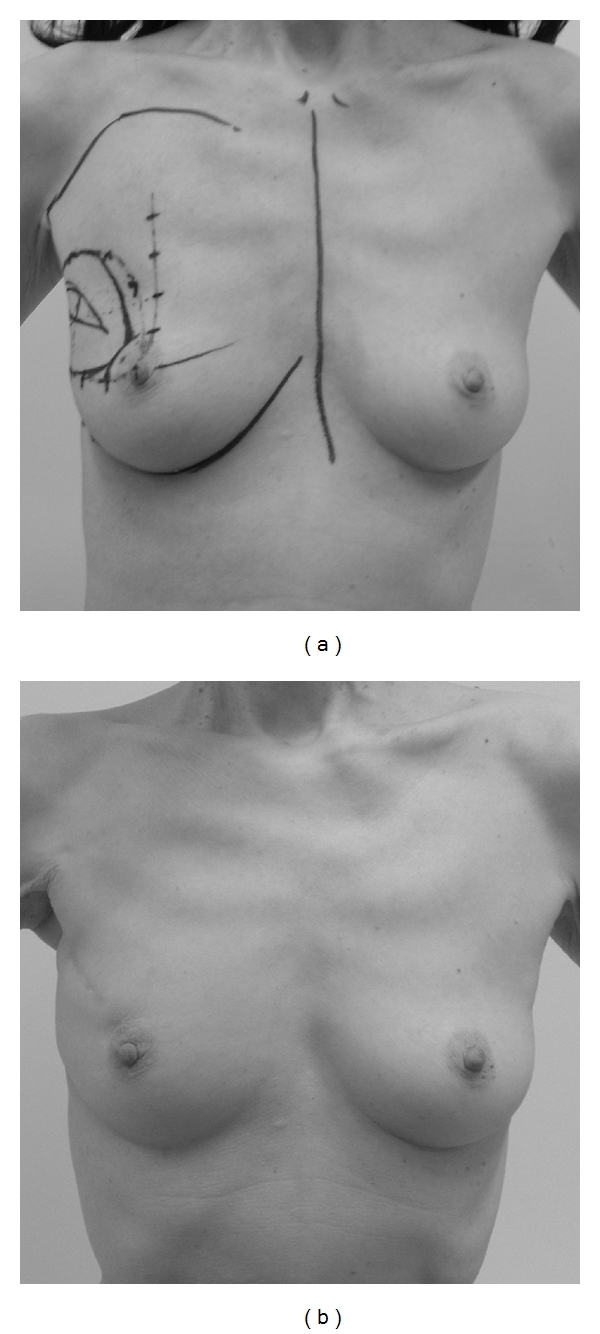
A case with excellent cosmetic results. The tumor location was in the upper outer quadrant in this case. She experienced postoperative bleeding and had surgery again for bleeding and hematoma evacuation. (a) Preoperative design. (b) Photograph at 1 year 4 months after the operation.

**Figure 6:**
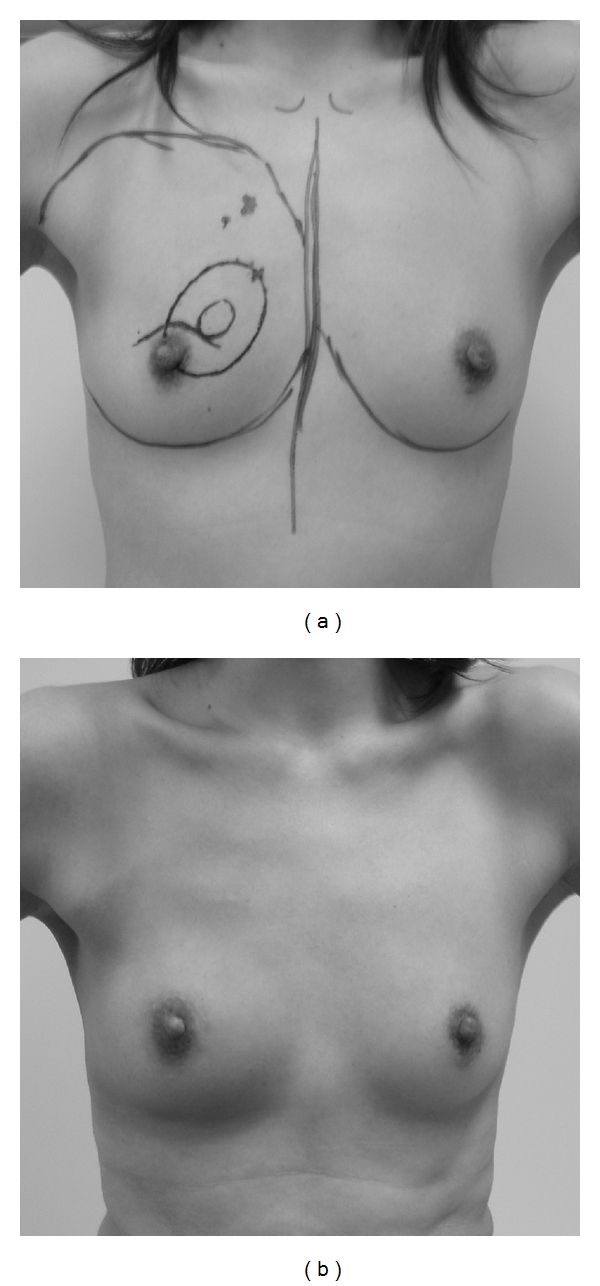
A case with excellent cosmetic results. The tumor location was in the upper inner quadrant in this case. (a) Preoperative design. (b) Photograph at 1 year after operation.

**Figure 7:**
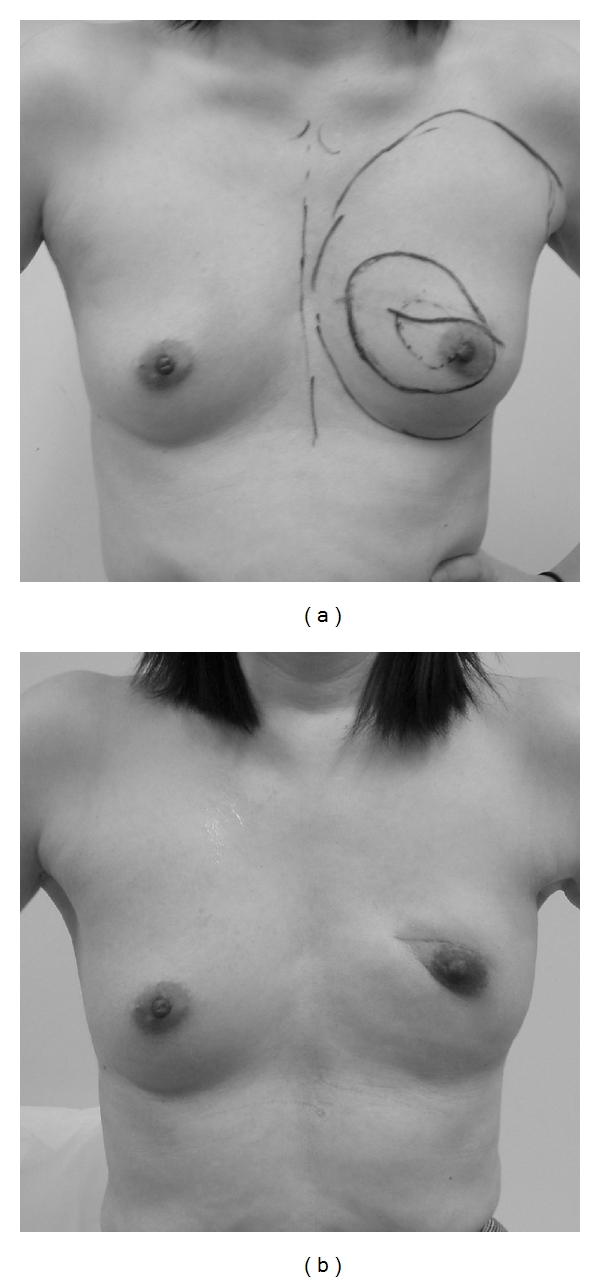
A case with poor cosmetic results. The tumor location was in the upper inner quadrant in this case, however, the resection area is included the lower inner quadrant. She had partial fat necrosis of the extended glandular flap. (a) Preoperative design. (b) Photograph at 1 year 2 months after operation.

**Figure 8:**
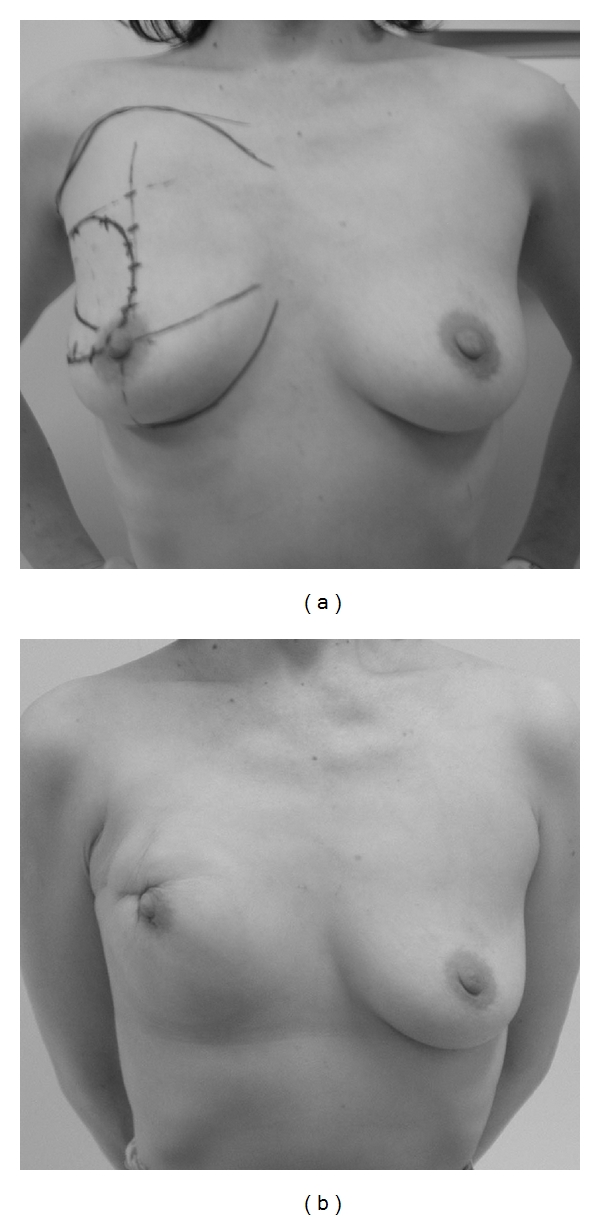
A case with poor cosmetic results. The patient experienced postoperative bleeding and hematoma. (a) Preoperative design. (b) Photograph at 1 year 6 months after operation.

 The cosmetic results at 1 year after operation of the 17 patients were assessed using photographs. The results were found to be excellent (Figure 5: Case  11, Figure 6: Case  2) in 11 cases (64.7%), good in 1 case (5.9%), fair in 3 cases (17.6%), and poor (Figure 7: Case  8, Figure 8: Case  15) in 2 cases (11.8%). An unacceptable outcome (either fair or poor) was seen in 5 cases (29.4%), and all cases except one were cases with complications (2 cases of postoperative bleeding and 2 cases of partial fat necrosis of the glandular flap). However the cosmetic result of the patient having surgery again for postoperative bleeding and hematoma was excellent (Figure 5: Case  11). The total follow-up period was 19 to 35 months after surgery (average 26.1 months), and there was no local recurrence. There were no changes in the cosmetic results over one year after the surgery had passed in patients with small-sized breast. In those with moderate or large breasts, however, one excellent case exhibited reduced breast volume more than one year after the surgery, and the cosmetic result at 2 years after the operation was downgraded to good.

## 4. Discussion

A number of clinical studies of early breast cancer have shown the advantages of breast-conserving surgery with an improved body image and a diminished psychological morbidity [6, 7]. However, the resection of large volumes of tissue can result in significant deformity [2, 8, 9]. The widespread popularity of breast-conserving surgery has generally focused attention on new oncoplastic techniques that can avoid unacceptable cosmetic results. Resection defects can be reconstructed in one of two ways: (i) by volume replacement, importing volume from elsewhere to replace the amount of tissue resected; or (ii) by volume displacement, recruiting and transposing a local tissue flap into the resection site. 

Many oncoplastic techniques have been reported, and some algorithms for selecting which oncoplastic technique to use for repairing partial mastectomy defects have been reported [1, 10–14]. Oncoplastic volume displacement techniques are best suited for Westerners who have large-sized breasts. Of course, if the defect is small, reconstruction by local tissue rearrangement is possible, regardless of the size of the breast. However, for larger defects in small breasts, reconstruction with volume displacement is not suitable because of the inadequate tissue available for performing a repair, so volume replacement reconstruction is usually recommended [13, 14]. 

A glandular flap is usually used for the filling of defects comprising less than 20% of the breast volume [1]. Our extended glandular flap is a type of glandular advancement flap, however, this flap can be used for replacement of a defect of more than 20% of the breast volume by including adipose tissue from the subclavicular area. Even if a defect is more than 20% in a small breast, the good cosmetic result can be obtained using this technique. 

Japanese women's breasts do not tend to be very large. On the other hand, some Japanese women have dense glandular breasts. A dense glandular breast can be easily mobilized by advancing the breast tissue into the excision cavity without a risk of necrosis [1]. Moreover clothing that shows the décolletage is popular in European country, so women want to avoide any deformity of the subclavicular area, however, such clothing is not popular in Japan. So it is possible for us to use adipose tissue from the subclavicular area for breast remodeling. Therefore, this extended glandular flap is often suitable for Japanese females. 

As reported herein, this surgical technique is particularly useful for patients with small dense breasts. For such breasts, this technique demonstrated good cosmetic results if the patients had no complications, such as postoperative bleeding and fat necrosis of the glandular flap. A reoperation should therefore be considered for postoperative bleeding, if drainage is difficult, because prolonged hematoma is considered to be the most adverse effect for cosmetic results.

On the other hand, moderate or large breasts are considered unsuitable for this technique, because the breast volume tends to decrease over time. As shown in Table 1, when there was more than 30% resection in moderate- or large-sized breasts, excellent cosmetic results could not be obtained. Therefore, other volume displacement techniques, such as therapeutic reduction mammoplasty, are considered more suitable for these patients. In one case that developed fat necrosis in a small breast (Figure 7: Case  8), the resection area also included the lower inner quadrant, so other surgical procedure should have been selected in this case.

It is very important to keep the perforators of the internal mammary artery and/or branches of the lateral thoracic artery intact while making the flap. This flap is similar to a perforator flap. In general, plastic surgeons tend to worry a lot about the blood supply, but some breast surgeons do not take this sufficiently into account to that. This procedure is easy to perform, thus making it possible for breast surgeons to perform it without the help of plastic surgeons. However, breast surgeons have to behave like plastic surgeons. 

Although the present study had a small sample of 17 cases, we were able to demonstrate the usefulness of breast-volume displacement using an extended glandular flap when performing breast conservation therapy for breast cancer occurring in the upper portion of small dense breasts. 

In addition, in the future, we would like to examine this technique in cases that require resection of more than 20% of small and dense breasts, because patients with a moderate or large breast size were found to be unsuitable for this technique. 

## Figures and Tables

**Table 1:** Summary of patients.

No.	Location	Age	Operation	Breast size	Excision size	%^(1)^	Breast density on MMG^(2)^	Suction drain	Hospital stay	Postoperative complication	Cosmetic result^(3)^
1	CD	65	Bp + SNB, Ax	Small	7.2 × 6.8 cm	30%	Heterogeneously dense	10 days	14 days	Delayed wound healing	Excellent
2	A	41	Bq + SNB	Small	7.5 × 6.7 cm	30%	Heterogeneously dense	2 days	6 days		Excellent
3	C	58	Bq + SNB	Small	7 × 5 cm	30%	Heterogeneously dense	3 days	7 days		Excellent
4	CD	36	Bq + Ax	Small	7.5 ×7.5 cm	30%	Heterogeneously dense	8 days	11 days		Excellent
5	EAC	43	Bp + SNB	Small	8 × 7 cm	30%	Heterogeneously dense	3 days	9 days		Excellent
6	A	38	Bq + SNB	Small	8 × 7 cm	30%	Heterogeneously dense	3 days	6 days		Good
7	CD	44	Bq + SNB	Small	9.5 × 7 cm	30%	Heterogeneously dense	3 days	6 days	Bleeding	Fair
8	AB	48	Bq + SNB	Small	9.5 × 9.2 cm	30%	Heterogeneously dense	3 days	7 days	Fat necrosis	Poor
9	C	44	Bq + SNB	Small	11 × 9 cm	40%	Heterogeneously dense	4 days	7 days	Delayed wound healing	Excellent
10	C	42	Bq + SNB, Ax	Small	13 × 10 cm	40%	Heterogeneously dense	6 days	10 days		Excellent
11	C	56	Bq + SNB, Ax	Small	15 × 9 cm	40%	Heterogeneously dense	10 days	14 days	Bleeding (re-operation)	Excellent
12	CD	60	Bp + SNB	Moderate	6.5 × 6.5 cm	20%	Heterogeneously dense	5 days	8 days		Excellent
13	CD	54	Bq + Ax	Moderate	12 × 8 cm	30%	Heterogeneously dense	10 days	14 days		Excellent*
14	C	46	Bq + SNB	Moderate	13 × 9 cm	40%	Heterogeneously dense	4 days	9 days		Fair
15	C	40	Bq + SNB	Moderate	14 × 8.5 cm	40%	Heterogeneously dense	10 days	13 days	Bleeding	Poor
16	A	36	Bq + SNB	Moderate	14 × 11.5 cm	40%	Heterogeneously dense	2 days	7 days	Fat necrosis	Fair
17	C	30	Bq + SNB	Large	10 ×10 cm	20%	Heterogeneously dense	4 days	8 days		Excellent

^(1)^Excision volume compared to the total breast volume was estimated by using the photograph of preoperative marking of a partial resection area.

^(2)^Breast density was classified into four categories based on the Breast Imaging Reporting and Data System (BI-RADS).

^(3)^The cosmetic result was evaluated based on photographs taken one year after the operation.

*The cosmetic result of 2 years after the operation was down to be good.
